# Assessing the toxicity of one-step-synthesized PEG-coated gold nanoparticles: *in vitro* and *in vivo* studies

**DOI:** 10.31744/einstein_journal/2024AO0764

**Published:** 2024-05-06

**Authors:** Murilo Montenegro Garrigós, Fernando Anselmo de Oliveira, Cícero Júlio Silva Costa, Lucas Renan Rodrigues, Mariana Penteado Nucci, Arielly da Hora Alves, Javier Bustamante Mamani, Gabriel Nery de Albuquerque Rego, Juan Matheus Munoz, Lionel Fernel Gamarra

**Affiliations:** 1 Hospital Israelita Albert Einstein São Paulo SP Brazil Hospital Israelita Albert Einstein, São Paulo, SP, Brazil.; 2 Hospital das Clínicas Faculdade Medicina Universidade de São Paulo São Paulo SP Brazil LIM44 - Hospital das Clínicas, Faculdade Medicina, Universidade de São Paulo, São Paulo, SP, Brazil.

**Keywords:** Nanomedicine, Flow cytometry, *In vitro* techniques, Nanoparticles, polyethylene glycols, Toxicity

## Abstract

**Objective:**

To evaluate the *in vitro* and *in vivo* toxicities of polyethylene glycol-coated gold nanoparticles synthesized using a one-step process.

**Methods:**

Gold nanoparticles were prepared via a co-precipitation method using polyethylene glycol, and the synthesis product was characterized. For the *in vitro* evaluation, a flow cytometry analysis with Annexin V and iodide propidium staining was used to assess cytotoxicity in MG-63 cells labeled with 10, 50, and 100µg/mL of nanoparticle concentration. For the *in vivo* evaluation, nanoparticles were administered intraperitoneally at a dose of 10mg/kg dose in 10-week-old mice. Toxicity was assessed 24 hours and 7 days after administration via histopathological analysis of various tissues, as well as through renal, hepatic, and hematopoietic evaluations.

**Results:**

Synthesized nanoparticles exhibited different hydrodynamic sizes depending on the medium: 51.27±1.62nm in water and 268.12±28.45nm (0 hour) in culture medium. They demonstrated a maximum absorbance at 520nm and a zeta potential of -8.419mV. Cellular viability exceeded 90%, with less than 3% early apoptosis, 6% late apoptosis, and 1% necrosis across all labeling conditions, indicating minimal cytotoxicity differences. Histopathological analysis highlighted the accumulation of nanoparticles in the mesentery; however, no lesions or visible agglomeration was observed in the remaining tissues. Renal, hepatic, and hematopoietic analyses showed no significant differences at any time point.

**Conclusion:**

Polyethylene glycol-coated gold nanoparticles exhibit extremely low toxicity and high biocompatibility, showing promise for future studies.

## In Brief

We demonstrated that one-step-synthesized PEG-coated gold nanoparticles have high stability, very low toxicity, and high biocompatibility in human cells and animal models. These properties make them promising candidates for use in future studies as diagnostic tools, therapeutic agents, drug delivery systems, and in many other medical applications.

## Highlights

O۪۪ne-step synthesis provides affordable, stable, and biocompatible nanoparticles.

P۪۪EG-coated gold nanoparticles exhibited very low cytotoxicity effects.

T۪۪his in vivo study did not reveal hematopoietic, renal, or hepatic alterations.

T۪۪he histopathological analysis did not present any tissue or cellular damage.

## Graphical abstract

**Figure:**
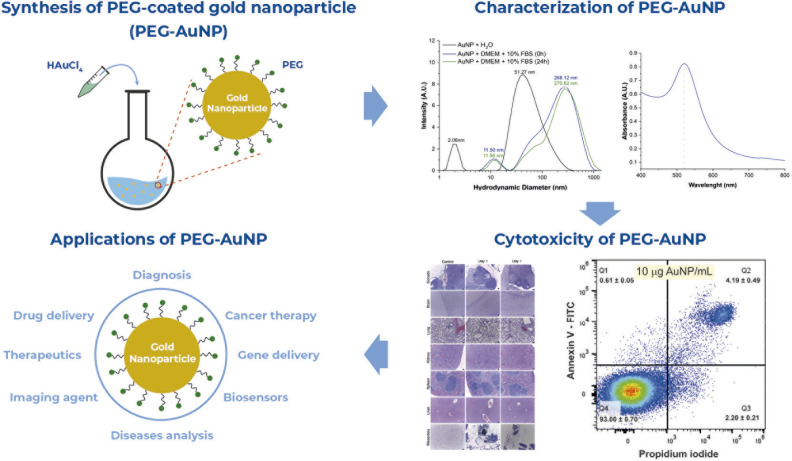

## INTRODUCTION

Significant attention has been devoted to the development of nanomaterials for disease diagnosis, therapy, and prevention.^(1)^ The use of biocompatible nanoparticles (NPs) for disease detection, drug delivery systems, or as alternative therapies in living organisms has garnered attention for its ability to address various challenges inherent in other methods.^(2,3)^ These challenges include the effective targeting pharmacological agents to tumors and overcoming issues associated with tumors receiving low concentrations of drugs.^(4-6)^ Despite being advantageous, one of the greatest challenges related to the use of NPs is the development of biocompatible materials that have low toxicity and are capable of internalization in the organism without presenting unwanted effects on cell viability and tissue structure.^(7-9)^

In this context, gold nanoparticles (AuNPs) are extremely promising.^(10)^ In addition to being stable, simple to manufacture, controllable in size, and straightforward for surface functionalization, these NPs also present strongly enhanced optical properties and high biocompatibility for *in vivo* studies.^(11)^ The utilization of AuNPs has thus distinguished itself in a number of applications, serving as promising carriers for different biomolecules, such as drugs, therapeutic proteins, DNA, or RNA,^(12,13)^ in addition to acting as phototherapeutic and tumor detection agents.^(13-16)^

One of the more feasible options for enhancing the medical properties of AuNPs is PEGylation, which involves coating NPs with PEG, a biopolymer that is affordable, versatile, and FDA-cleared.^(17)^ PEG-coated AuNPs (PEG-AuNPs) offer improved cellular uptake, lower cytotoxicity, and mitigation of adverse immune system responses compared to AuNPs typically coated with citrate. Moreover, PEGlyation allows for easier passage of NPs through different biological barriers, such as the blood-brain barrier.^(12,18)^

One current challenge in nanomedicine is the development of simple, rapid, biocompatible, and affordable PEGylation methods.^(10)^ The use of unmodified PEG as a reducing and stabilizing agent for AuNPs has been shown to be a viable alternative because it is capable of synthesizing stable and low-polydispersity NPs via a controllable and reproducible one-step synthesis process.^(19)^

We evaluated the cytotoxicity of PEGAuNPs in fibroblast-like cells via flow cytometry and *in vivo* toxicity via histopathological analysis of various tissues. We also conducted hematological, hepatic, and renal evaluations of C57BL/6 mice 1 and 7 days postadministration.

## OBJECTIVE

Based on this, the current study aimed to evaluate the *in vitro* and *in vivo* toxicity of PEG-AuNP synthesized through a simple one-step synthesis process.

## METHODS

### *In vitro* studies

#### Synthesis of PEG-AuNPs

PEG-AuNPs were synthesized using a one-step process, according to Stiufiuc et al.^(19)^ Briefly, the procedure was performed in a 150mL single-neck round-bottomed flask containing a solution of 45mL of MilliQ^®^ water (EMD Millipore Corporation, MA, USA), 6g of PEG1500 (Sigma-Aldrich, MO, USA), and 0.75mL NaOH 1%m/m solution (Merck, Darmstadt, Germany). Subsequently, the system was heated to 50°C under magnetic stirring. A 5mL aqueous solution containing 19.7mg of HAuCl_4_ (Sigma-Aldrich, MO, USA) was then added to the flask, and the resulting transparent solution was heated up to 80°C under stirring. Once a characteristic ruby-red color developed, the solution was stirred and heated at 80°C for an additional 3 minutes before suspending the reaction. The product was then allowed to cool to room temperature.

#### Characterization of PEG-AuNPs

To evaluate the hydrodynamic diameter of the synthesized PEG-AuNPs, a solution of 50µg/mL was prepared for dynamic light scattering (DLS) analysis using a Zetasizer Ultra System (Malvern, Worcestershire, UK), employing the same parameters reported in a previous study.^(20)^ Dulbecco’s Modified Eagle’s Medium/F12 (DMEM) supplemented with 10% fetal bovine serum (FBS) was used, with a viscosity of 0.94 centipoise. The mean diameter and standard deviation were obtained by fitting the experimental data to a lognormal distribution function. To verify the effects of the culture medium on the polydispersion and stability of NPs (0 and 24 hours), the same DLS conditions were used for a solution of PEG-AuNP resuspended in DMEM (GIBCO^®^ Invitrogen Technologies, NY, USA) and supplemented with 10% FBS (GIBCO^®^ Invitrogen Technologies, NY, USA).

The zeta potential was immediately evaluated using the Zetasizer Ultra System with a 50µg/mL PEG-AuNP solution at pH 7.4 and 37°C. Subsequently, the surface plasmon resonance (SPR) of the prepared NPs was determined by obtaining the absorption spectrum of a 50µg/mL PEG-AuNP solution ranging from 400 to 800nm. Absorption spectra were obtained using a UV-1800 spectrometer (Shimadzu, Kyoto, Japan).

#### Cell culture of MG-63 lineage

To further evaluate the cytotoxicity of the synthesized PEG-AuNPs, we used the fibroblast-like MG-63 cell line obtained from the Cell Bank of Rio de Janeiro (BCRJ, code: 0173). The cells were cultivated using DMEM supplemented with 10% FBS, 1% of penicillin-streptomycin (GIBCO^®^ Invitrogen Corporation, CA, USA), and 1% of L-glutamine (GIBCO^®^ Invitrogen Corporation, CA, USA) and incubated at 37°C, 5% CO_2_, and 60% relative humidity. For detachment of the MG-63 cells from the T-25 flasks, 0.25% trypsin EDTA (GIBCO^®^ Invitrogen Corporation, CA, USA) was added at 37°C, followed by a 5-minutes incubation period in a 5% CO_2_, 98% of humid atmosphere at 37°C.

#### Evaluation of PEG-AuNP cytotoxicity on MG-63 cells

The evaluation of PEG-AuNP cytotoxicity was performed using a fluorescein isothiocyanate (FITC) Annexin V Apoptosis Detection Kit 1 (BD Biosciences, CA, USA), followed by flow cytometry on an Attune™ NxT Flow Cytometer (Thermo Fisher Scientific, MA, USA). Briefly, MG-63 cells were seeded in a 24-well plate at a density of 1 × 10^5^ cells per well. After 48 hours, the culture medium was replaced, and the cells were labeled with 10, 50, and 100µg/mL of PEG-AuNP for 24 hours. Cells were detached via trypsinization and resuspended in DMEM. Cytotoxicity was evaluated using FITC-Annexin V and propidium iodide staining kits (Thermo Fisher Scientific, Eugene, EUA), according to the manufacturer’s instructions. The data obtained were analyzed using FlowJo software version 10.6 (BD Biosciences, CA, USA), where the cells were categorized as early apoptotic (Annexin V+/PI-), late apoptotic (Annexin V+/PI+), necrotic (Annexin V-/PI+), or viable (Annexin V-/PI-).

## *In vivo* studies

### Ethics statement

This study was approved by the Ethics in Animal Research Committee of *Hospital Israelita Albert Einstein* (number 4932/21). Twenty-four 10-week-old female C57BL/6 mice were used. These animals were maintained at 21±2°C and 60%±5% relative humidity with full ventilation, under a 12-hours light/dark cycle (from 7 am until 7 pm). All mice had access to food and water *ad libitum* at the *Centro de Experimentação e Treinamento em Cirurgia*, a vivarium accredited by the Association for the Assessment and Accreditation of Laboratory Animal Care International (AAALAC International).

### PEG-AuNP *in vivo* administration

To avoid the contamination and administration of eventual clusters of NPs, the PEG-AuNPs were filtered through a 0.22µm sterile syringe filter. The material was then administered intraperitoneally at a final dose of 10mg/kg. After 1 and 7 days of administration, the animals were euthanized via anesthesia overdose to collect organs (gonads, brain, lung, kidney, spleen, liver, and mesentery) for histopathological analysis.

### Histopathological analysis

Histopathological analysis was performed using hematoxylin and eosin (H&E) staining to evaluate the possible effects and toxicity of PEG-AuNPs after 1 and 7 days of administration (n=5 per time of evaluation). All the tissues were initially fixed in flasks containing a 4% buffered paraformaldehyde solution. Subsequently, the samples were dehydrated in absolute alcohol for 5 hours, diaphonized in xylene for 3 hours, and immersed in paraffin for 2 hours at 60°C. The blocks were sliced to a thickness of 5µm using a Leica RM2245 microtome (Leica, IL, USA) and stained using the H&E standard protocol. Microscopic examination was performed using a Nikon TiE fluorescence microscope (Nikon, Tokyo, Japan).

### Blood count and biochemical analysis

To evaluate the influence of PEG-AuNPs on the hematopoietic environment and the kidney and liver systems, blood samples were collected from each animal (n=9). Serological analysis was performed using a ChemWell T Auto-Analyzer (Awareness Technology, FL, USA) for alanine transaminase (ALT), aspartate transaminase (AST), albumin, blood urea nitrogen (BUN), and creatinine. Subsequently, the blood count analysis was performed using a Hematoclin 2.8 vet (Bioclin, Belo Horizonte, Brazil) for granulocytes, lymphocytes, red blood cells (RBCs), and platelets.

### Statistical analysis

The data were described as the mean and standard deviation. Inferential statistical analysis was conducted using the one-way analysis of variance (ANOVA) test for cytotoxicity evaluation and blood analyses. Post hoc analysis was performed using the Bonferroni correction method. Statistical analyses were performed using JASP software v0.14.1 (http://www.jasp-stats.org; accessed on July 14, 2023). The statistical significance level was set at p<0.05.

## RESULTS

### Characterization of PEG-AuNPs

**Figure 1:**
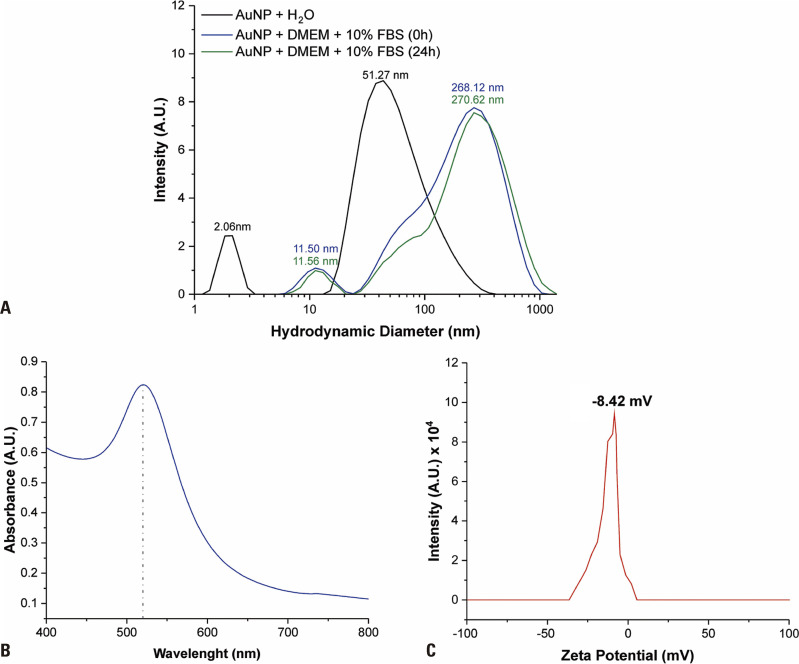
AuNPs: gold nanoparticles; DMEM: Dulbecco’s Modified Eagle’s Medium/F12; FBS: fetal bovine serum. . Characterization of PEG-AuNPs. (A) Hydrodynamic diameter size evaluation via dynamic light scattering of PEG-AuNPs in water and culture media; (B) PEG-AuNP absorption spectrum; (C) zeta potential measurements of PEG-AuNPs

The hydrodynamic diameter and optical and surface charge characterizations of the synthesized PEG-AuNPs were performed using DLS, UV-VIS, and zeta potential techniques, as depicted in figure 1 (parts A, B, and C, respectively).

Figure 1A shows the DLS measures obtained from PEG-AuNPs, along with potential interferences from the culture medium. Readings were performed in MilliQ^®^ water and in DMEM supplemented with 10% of FBS. Two peaks with different sizes and intensities were observed for each condition. In MilliQ^®^ water, a more intense peak was observed at 51.27±1.62nm, attributed to PEG-AuNPs, and another at 2.06±0.01nm, representing residual PEG in solution. Conversely, in the culture medium, the peaks were less intense but exhibited larger hydrodynamic diameters. Specifically, PEG-AuNPs showed a size of 268.12±28.45nm (0 hour) and 270.62±22.17nm (24 hours), while the PEG peak shifted to 11.50±0.05nm (0 hour) and 11.56±0.04nm (24 hours), showing adequate stability over 24 hours, consistent with the duration of the cytotoxicity evaluation.

The optical properties of PEG-AuNPs, as analyzed by the absorbance spectrum (Figure 1B), revealed a maximum absorbance at 520nm. Additionally, the value of the zeta potential of the synthesized PEG-AuNPs was determined to be approximately -8.42mV, as shown in figure 1C.

### Cytotoxicity of PEG-AuNPs for MG-63 cells

**Figure 2:**
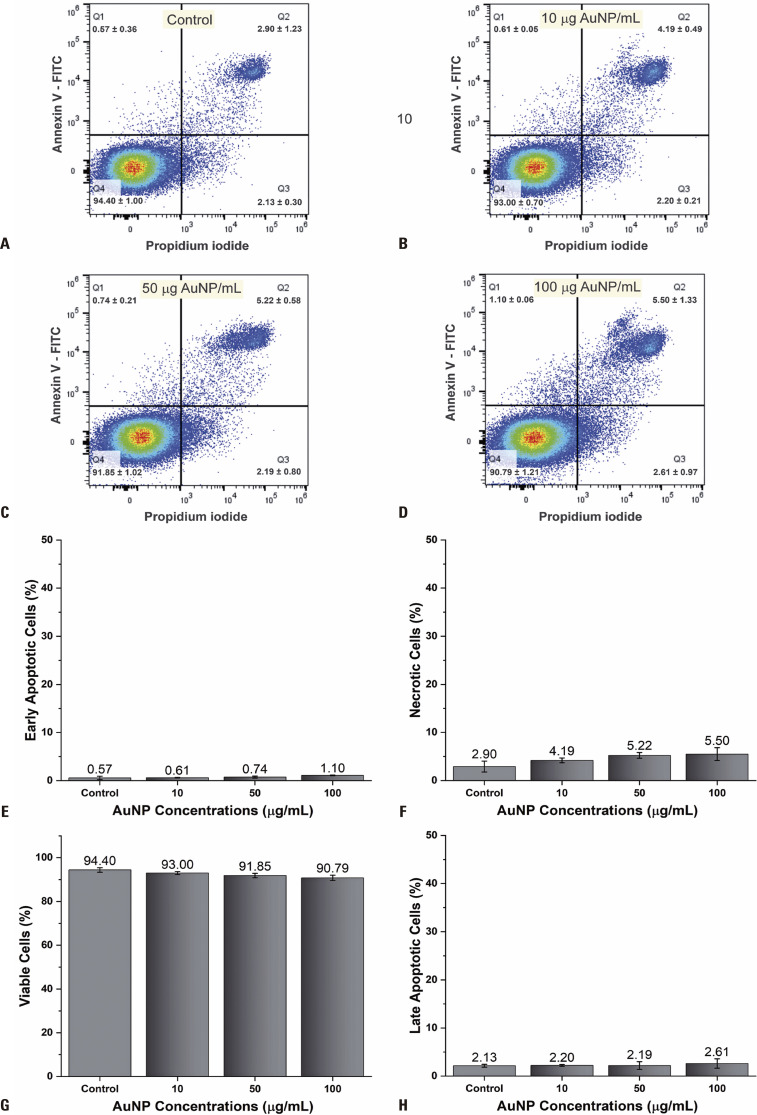
PEG-AuNP cytotoxicity evaluation via flow cytometry. (A–D) Representative flow cytometry charts of cell labeling with 0 (control), 10, 50, and 100µg/mL of PEG-AuNPs, respectively; (E–H) Analysis of quadruplicate samples of the percentage of early apoptotic, necrotic, viable, and late apoptotic cells, respectively

Flow cytometry was used to analyze the MG-63 cell toxicity for all PEG-AuNP concentrations tested (control, 10, 50, and 100g/mL) in the cellular labeling process, as shown in figure 2 (A to D). The cellular condition corresponding to increasing NP concentrations in the labeling process was depicted by the quadrants, where Q1 represented early apoptosis cells ranging from 0.57±0.36 to 1.10±0.06, Q2 denoted necrotic cells (2.90±1.23 to 5.50±1.33), Q3 indicated late apoptosis cells ranging from 2.13±0.30 to 2.61±0.97, and Q4 depicted viable cells ranging from 94.40±1.0 to 90.79±1.21.

The quadruplicate sample results of this assay were plotted in the histogram by percentile values of each cell condition (Figure 2 E to H), with no significant difference being observed with increased PEG-AuNP concentration for each cell condition using ANOVA. Low cell toxicity was observed at higher NP concentrations (90.79%) compared to the control (94.40%), with evident dose-dependent toxicity (necrotic cells increased from 2.90% to 5.50% with an increase in NP concentration; figure 2F; the percentage of early and late apoptotic cells was less than 1.1% and 2.6%, respectively, with small variations between labeling conditions. With the control representing 100% viability, the other conditions tested exhibited the following percentages of viability: 98.52% (10µg/mL), 97.30% (50µg/mL), and 96.18% (100µg/mL). Therefore, the higher concentration used for cellular labeling only decreased cell viability by 3.82%.

### *In vivo* evaluation of PEG-AuNP toxicity

#### Histopathological analysis

**Figure 3:**
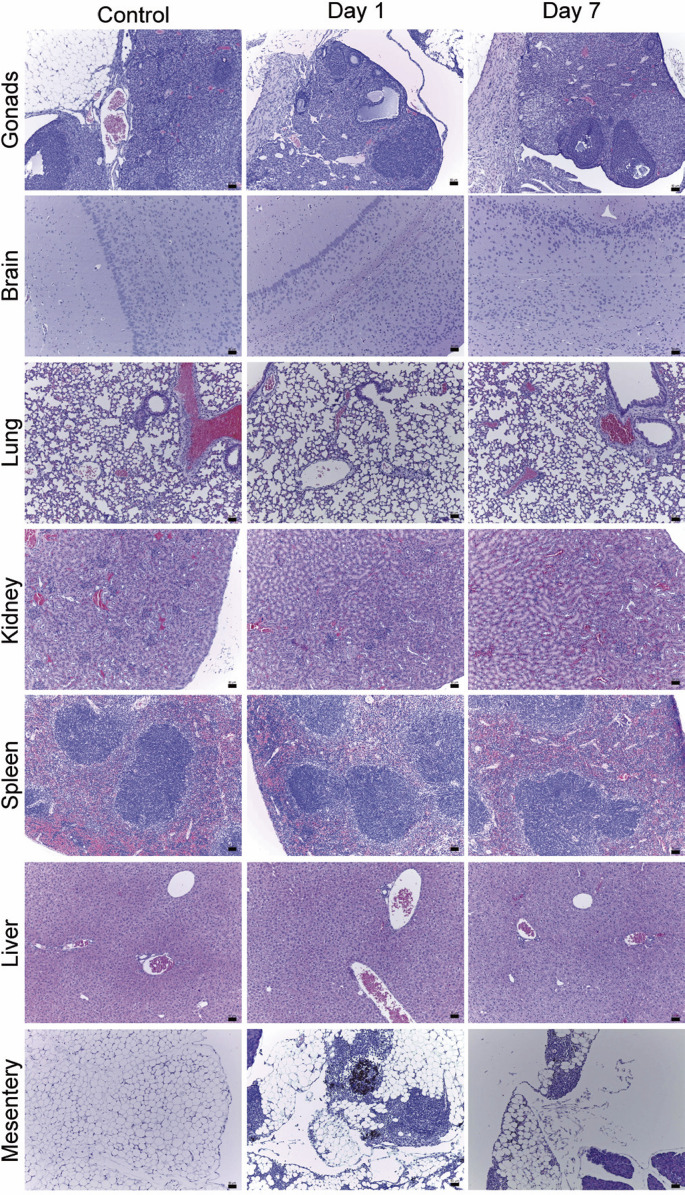
Histopathological evaluation of the gonads, brain, lung, kidney, spleen, liver, and mesentery of control animals and animals after 1 and 7 days of PEG-AuNP administration

Histopathological analysis (Figure 3) revealed no tissue or cellular damage attributable to PEG-AuNP toxicity in the animal models across the main tissues of the evaluated organs over time. However, PEG-AuNP agglomeration was observed in the mesentery on the first day, with a subsequent decrease in extent by the seventh day. Signs of inflammation were also observed at this site, with an increase in the macrophage population.

#### Blood count and biochemical analysis

**Figure 4:**
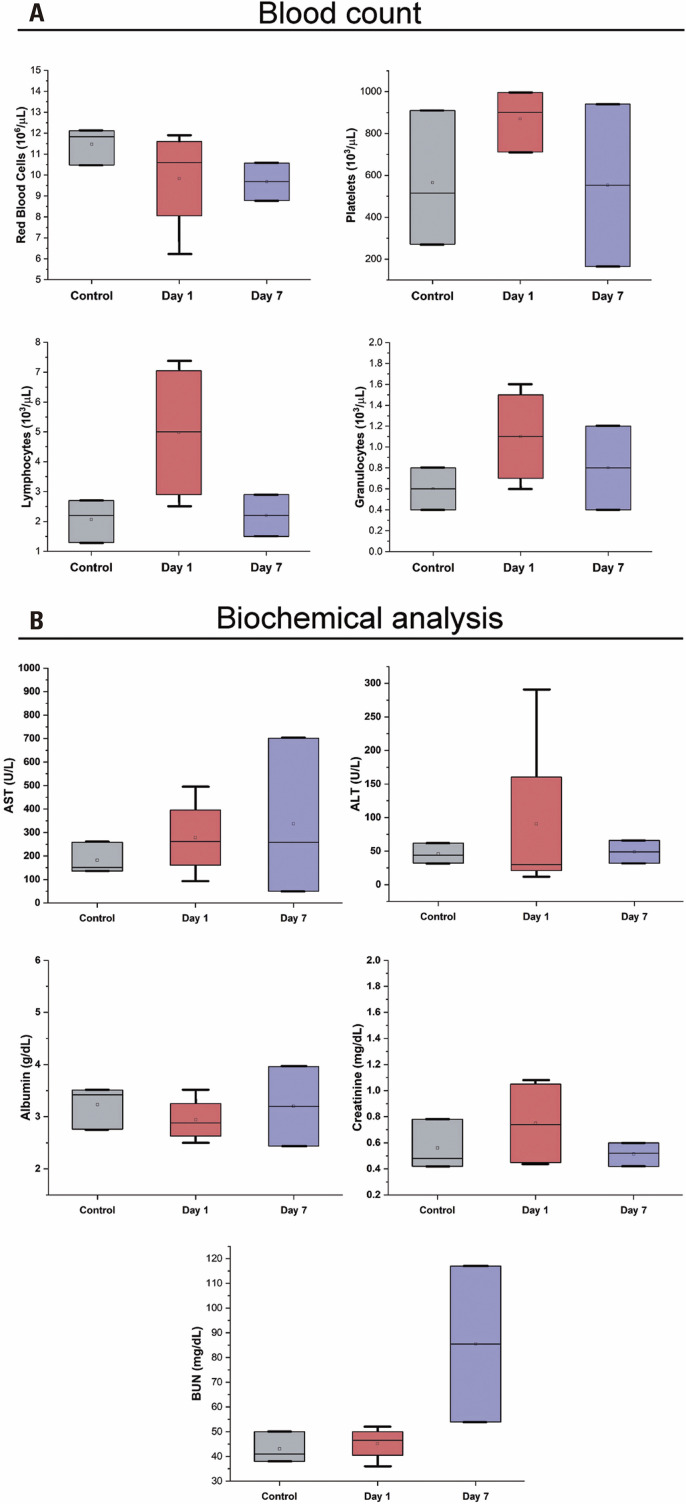
Biochemical and blood count analyses. (A) Hematological evaluation for platelets, red blood cells, lymphocytes, and granulocytes; (B) Biochemical analyses of aspartate transaminase, alanine transaminase, albumin, creatinine, and blood urea nitrogen

There were no significant differences in the RBC, platelet, lymphocyte, or granulocyte populations over time (Figure 4A). Moreover, no differences in AST, ALT, or albumin levels were observed over time in the hepatic evaluation (Figure 4B). Renal evaluation showed an increase in BUN values (p=0.154) between 1 and 7 days post-administration, whereas creatinine levels remained similar throughout the entire experiment (Figure 4B). These results indicate that PEG-AuNPs did not affect the hematopoietic, hepatic, or renal systems, corroborating the histopathological findings and reinforcing PEG-AuNP biocompatibility.

## DISCUSSION

This study demonstrated that PEG-AuNPs synthesized using a one-step protocol demonstrate sufficient properties for biomedical applications. PEG-AuNPs were evaluated *in vitro* and *in vivo* under various physiological conditions, exhibiting acceptable stability, adequate zeta potential, high biocompatibility, and low toxicity.

AuNPs find significant applications in nanomedicine, particularly in biosensor labeling, bioimaging, drug delivery systems, and phototherapeutic roles.^(12,13,15,21)^ Recent research have focused on the development of affordable biocompatible NPs that can be employed in clinical trials.^(7)^ In this scenario, AuNPs stand out because of their high biocompatibility and ability to be used in different applications.^(7,10,11)^ To improve the biocompatibility and the medical applications of AuNP, researchers have turned to coating these NPs with PEG, a biocompatible polymer^(22)^ that avoids non-specific binding with proteins. This contributes to the stability of NPs and improves their efficiency in the physiological environment.^(23,24)^ Moreover, various nanomedicine disciplines have focused on devising rapid and economical PEGylation methods, with one-step syntheses of AuNPs particularly praised for their simplicity and efficiency.^(19,25)^

In this study, we aimed to fill a gap in the literature on the toxicity of PEG-AuNPs synthesized using a one-step co-precipitation method that does not require the use of organic solvents or toxic reagents. This is in contrast to most one-step-synthesized AuNPs reported in the literature, which are synthesized using citrate to reduce Au(III) to Au(0).^(26,27)^ In our synthesis protocol, PEG acts as both a reducing agent and stabilizer,^(23)^ enabling a simple, fast, affordable, and direct application of these nanomaterials from the synthesis product.^(19,25,28)^ This approach aims to improve the viability of using the nanomaterial in both clinical and pre-clinical studies.

It is necessary to characterize this material prior to its application in toxicity tests. This study showed that PEG-AuNPs achieved adequate properties post-synthesis and were stable under physiological labeling conditions (*in vitro*: water and medium culture) over 24 hours. Despite showing an increase in size and in corona protein formation due to interactions between NPs and complex components of the culture medium – driven by electrostatic forces, van der Waals forces, steric forces, and magnetic forces modulated by the Brownian motion associated with NPs^(29,30)^ – PEG-AuNPs maintain colloidal stability and resist aggregation, consistent with Moore et al.^(31)^ The absorbance spectrum displayed the typical SPR peak of 50nm AuNPs.^(32)^ Lastly, the zeta potential indicated a low surface charge and efficient PEGylation.^(19,33)^

The labeling of MG-63 cells with AuNPs in previous studies^(34,35)^ reported no significant effects on the cellular cycle or key signaling pathways after labeling with up to 40µM AuNPs, consistent with our results. Steckiewicz et al.^(36)^ reported that high cell viability exceeding 95% was observed in MG-63 cell labeling with concentrations of up to 5µg/mL of spherical AuNPs, whereas our study demonstrated 96.18% viability even at concentrations of up to 100µg/mL, representing a mere 3.82% compromise in viability. This indicates high biocompatibility and low cytotoxicity of PEG-AuNPs.

The toxicity evaluation of PEG-AuNPs *in vivo* was evaluated via histopathological analysis, biochemical evaluation of renal and hepatic parameters, and blood count. Histopathological analysis highlighted an accumulation of PEG-AuNPs at the mesentery site, probably because of the route of administration, as already observed by Pham et al.^(37)^ Moreover, the accumulation was reduced after 7 days, indicating that NPs progressively left this site over time. Signs of inflammation were also identified in the mesentery; however, as PEG-AuNPs are foreign to the body, certain degrees of inflammation are still considered a normal response, as well as an increase in the population of macrophages in the region of accumulation.^(38,39)^ Because PEG-AuNPs are distributed in other places in a more subdued and non-agglomerated manner than in the mesentery, no obvious agglomeration of PEG-AuNPs was observed in the analyses of the other tissues.^(40-44)^ The absence of substantial toxicity signals and the lack of significant lesions in the tissues indicate the material’s potential biocompatibility and low toxicity, both of which have been previously reported.^(45-47)^ The observation of inflammation solely in the mesentery further underscores the material’s low toxicity, as the inflammatory response was localized, relatively mild, and attributed to NP agglomeration resulting from the administration route.^(48)^

The histopathological findings corroborated with biochemical and blood count analyses, and no significant variation was observed over time, demonstrating that PEG-AuNPs exhibited no significant effects on the hepatic, renal, or hematopoietic systems, reaffirming the low toxicity already reported in previous studies.^(49-51)^

Further development of this study could involve assessing the biodistribution of PEG-AuNPs using other methods, such as inductively coupled plasma mass spectrometry.

## CONCLUSION

The *in vitro* and *in vivo* assessments of PEG-AuNPs, synthesized via a one-step synthesis method, demonstrated no toxic effects on MG-63 cells even at high labeling concentrations. Moreover, up to 7 days post-administration, no significant lesions were observed in tissues used for *in vivo* testing, with no indications of renal or hepatic function alterations. The lymphocyte, granulocyte, platelet, and red blood cells populations were unaffected by PEG-AuNPs. Therefore, these findings suggest the exceptional promise of these PEG-AuNPs for future investigations and potential translation into clinical studies, given their extraordinarily low toxicity and high biocompatibility.
